# Hepatitis E virus infection in Turkey: a systematic review

**DOI:** 10.1186/s12941-018-0269-6

**Published:** 2018-05-02

**Authors:** Hakan Leblebicioglu, Resat Ozaras

**Affiliations:** 1 Department of Infectious Diseases and Clinical Microbiology, Ondokuz Mayis University, Medical School, Samsun, Turkey; 2 Medilife Hospital, Istanbul, Turkey

**Keywords:** Hepatitis E virus, Turkey, Prevalence, Systematic review, Travel

## Abstract

Hepatitis E virus (HEV), a non-enveloped single stranded RNA virus causes sporadic cases of hepatitis or outbreaks. The disease is generally self-limited although it may cause fulminant hepatitis in pregnant women, elderly, those with underlying chronic hepatitis, immunosuppressed, and transplant recipients. It is transmitted through fecal–oral route and zoonotic transmission. Hepatitis is a main health care problem in Turkey; HBV and HCV prevalences are 4 and 1% respectively. Hepatitis D represents another considerable hepatitis etiology with a prevalence of 5–27%. The information about HEV is not clear. In this systematic review, we aimed to analyze HEV studies reported from Turkey, to determine the current situation of the disease in the country, to delineate the limits of the studies and to determine the future study areas. The prevalence of HEV ranged from 0 to 12.4%. Children had lower prevalence than the adults. The prevalence was determined as 7–8% in pregnant women, 13% in chronic HBV patients, 54% in chronic HCV patients, 13.9–20.6% in patients with chronic renal failure, and ≈ 35% in agriculture workers. Among individuals immigrating form Turkey to Europe, HEV seroprevalence was found 10.3% in Italy and 33.4% in the Netherlands. HEV prevalence seems high in certain risk groups. Although previous studies suggest that Turkey is among the endemic countries of HEV, there are some pitfalls for the analysis of data: the studies are not powered enough to represent the whole population; they did not include immunosuppressed patients and solid organ recipients; and the prevalence of non-A non-B hepatitis was not determined.

## Background

Hepatitis E virus (HEV) was first identified in 1983. It causes sporadic cases of hepatitis or outbreaks and the disease is generally self-limited although it may cause fulminant hepatitis in pregnant women, elderly, those with underlying chronic hepatitis, immunosuppressed, and transplant recipients [1, 2]. It is a non-enveloped single stranded RNA virus in the genus *Hepevirus* and the family Hepeviridae. It has four genotypes. Genotypes 1 and 2 cause disease in humans while genotypes 3 and 4 cause diseases both in humans and animals especially in pigs [3]. HEV can be transmitted waterborne, foodborne, or zoonotic. While fecal–oral route is common in the countries where HEV is endemic, in developing countries, zoonotic transmission is more prevalent and causes sporadic infections [4, 5]. Seroprevalence differs according to the way of transmission. According to World Health Organization (WHO), 20 million HEV infections develop every year, 15% of them being symptomatic [6].

Turkey is a developing country; annual income is 25,275 US$/capita, with a population of 77 million, surface area of 783.563 km^2^ and with 62.5% agricultural land [7]. Viral hepatitis is a challenging health problem with a significant morbidity. Hepatitis seroprevalence differs among regions probably due to the socio-economical differences. HBV and HCV prevalences are 4 and 1% respectively [8]. Hepatitis D represents another considerable hepatitis etiology with a prevalence of 5–27% [9].

Hepatitis A and hepatitis E are endemic in the country. The first study reported HEV seroprevalence as 5.9% in 1993 [10]. Limited number of epidemiological studies was published after that preliminary study. In this systematic review, we aimed to analyze HEV studies reported from Turkey, to determine the current situation of the disease in the country, to delineate the limits of the studies and to determine the future study areas.

## Methods

This systematic review was prepared according to the guideline of preparation and report of systematic review (PRISMA, Preferred Reporting Items for Systematic Reviews and Meta-Analyses) [11]. Three main health and biomedical databases of Pubmed, Scopus, and Science Citation Index (SCI) were used for the literature search. Since HEV was discovered in 1983 [1], the search period was taken as 1980 to June 2017.

The search was done using the terms of “Hepatitis E, hepatitis E virus, Turkey, Turkiye, Travel migrant” in the three databases in order to determine all publications about HEV from Turkey. The language was not restricted on the search. Duplicate publications, those not including HEV and/or Turkey, reviews and meeting abstracts were excluded. The result was recorded in Endnote program. Diagrams were produced according to the PRISMA guideline.

### Data analysis

Study date, publication date, authors, type of study, study field, sample size, and age groups were identified and presented as tables.

## Results

**Fig. 1:**
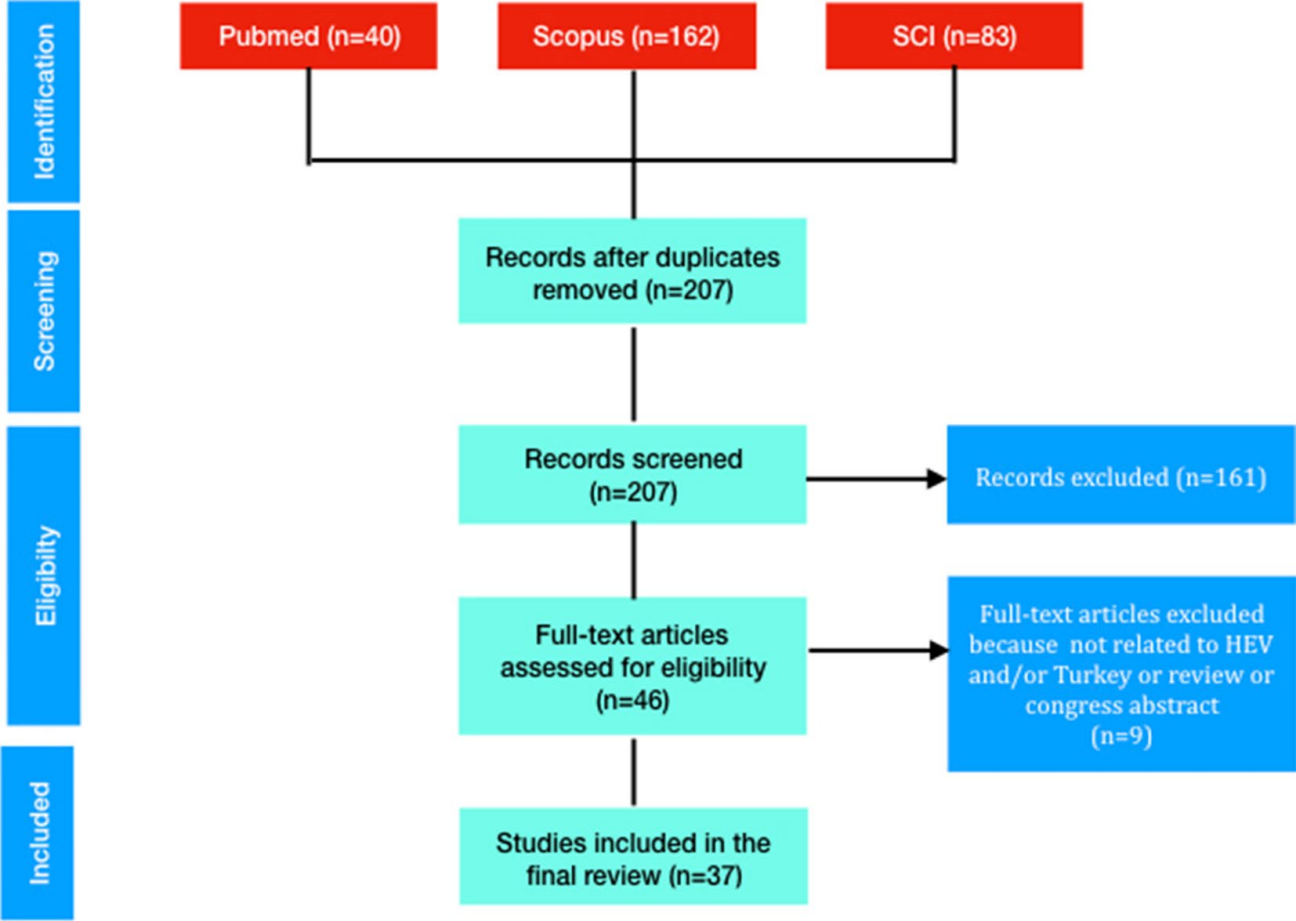
Flow diagram for literature search

The results of literature search were shown in flow diagram (Fig. 1). A total of 285 publications were identified in the databases; after removing duplicates, the abstracts of remaining 207 publications were further studied. Forty-six publications met the inclusion criteria. Another nine studies were noted not meeting the inclusion criteria after searching full texts and were excluded.

Among the remaining studies, one was a case report and another one investigated cupper level in patients with hepatitis including HEV. Twenty-eight publications were the seroprevalence studies in Turkey. Fifteen of these studies were in general population (Table 1), and 13 in specific groups: those with underlying disorders (n = 5), in patients presenting with acute hepatitis (n = 3), in pregnant women (n = 2), in those working in risky occupations (n = 2) and in those residing in the camps (n = 1) (Table 2).

**Table 1:** HEV seroprevalence studies in Turkey

Authors, references	Year published	Year	City	Study type	Sample size	Prevalence (IgG) (%)	Prevalence by age group	Power of study	Remarks
Aribas et al. [12]	2000	NA	Konya	C-S	162	12.3		NA	Children admitted to hospital
Atabek et al. [13]	2004	2001–2002	Konya	C-S	210	5.7	1–6 year: 0, 7–12 year: 6.8%, 13–18 year: 8.9%	NA	Rural 8.5%, urban 0.2%, p > 0.05
Aydın et al. [14]	2015	2012–2013	Ankara	C-S	1043	4.4	0–18 year: 0, 19–55 year: 30.4%, 56–90 year: 69.5%	NA	
Bayhan et al. [15]	2016	2014	Van	C-S	408	4.2	4.4% in 0–5 year, 3.4% in 6–13 year, 5.7% in 14–18 year	Calculated	Individuals admitting to hospital were compared in age groups: no difference.
Cesur et al. [16]	2002	2000–2001	Ankara	C-S	1046	3.8	15–30 year: 0, 30–45 year: 4.4%, 45–60 year: 6.6%, > 60 year: 7.4%	NA	15–75 year age group admitting to hospital
Cevahir et al. [17]	2013	NA	Denizli	C-S	185	12.4	7 year: 18.1%, 14 year: 6.6%	NA	Rural %13.1 vs. urban %11.7, p > 0.05. 7 year had higher prevalence than 6 year group
Colak et al. [18]	2002	1996–1997	Antalya	C-S	338	0.9	1–5 year: 0, 6–11 year: 1.6%	NA	No seropositives in preschool children
Eker et al. [19]	2009	2005	Edirne	C-S	582	2.4		Calculated	No difference in subgroups. Age range was not provided
Kaya et al. [20]	2008	2003	Düzce	C-S	589	0.3	6 month–12 year: 0, 13–17 year: 0.8%	NA	No seropositives in < 13 year-old
Maral et al. [21]	2009	2003–2005	Ankara	L	515	1.7–2.1		NA	6–14 year group. Same group re-studied 2 years later
Olcay et al. [22]	2003	2000	Ankara, Manisa, Diyarbakir	C-S	910	6.3	7–14 year: 1.6%, 15–24 year: 3.3%, 25–64 year: 8.2%, > 64 year: 10%	NA	Ankara 2.7%, Manisa 3.8%, Diyarbakir %11.7, significant. In Diyarbakir prevalence increased by age
Sidal et al. [23]	2001	1997–1998	Istanbul	C-S	909	2.1	6 month–2 year: 4.8%, 2–5 year: 3.1%, 5–10 year: 2.1%, 10–16 year: 0.3%	NA	
Thomas et al. [10]	1993	1990–1992	Istanbul, Aydin, Ayvalik, Adana, Trabzon	C-S	1350	5.9	11–20 year: 0, 21–30 year: 3.7%, 31–40 year: 9.1%, 41–50 year: 5.7%, 51–60 year: 8.7%, 61–70 year: 6.9%, 71–80 year: 11.1%	NA	Older age, HCV, being in Adana city were determined as risk factors
Yuce et al. [24]	1998	NA	Ankara	C-S	400	0	0 month–17 year: 0	NA	

*C-S* Cross-sectional, *L* longitudinal, *y* years old, *m* months old, *NA* not available

**Table 2:** HEV seroprevalence in special groups

Authors, references	Year published	Year	City	Study type	Target population	Sample size	Prevalence	CG sample size	CG prevalence (IgG) (%)	Power of study	Remarks
Aksu et al. [25]	1999	1996–1998	İzmir	C-S	Behcet’s disease	124	7%	51	8	NA	p > 0.05
Atabek et al. [26]	2003	NA	Konya	C-S	Diabetic children	63	6.3%	63	7.9	NA	p > 0.05
Aydin et al. [27]	2016	NA	Erzurum	C-S	Animal workers	103	35.9%	92	4.4	NA	p < 0.05. Most frequent in animal husbandry, poultry. No seropositivity in veterinaries
Bayram et al. [28]	2007	2004	Gaziantep	C-S	Adult CHB and CHC	364	CHB: 13.7%, CHC: 54%. HEV RNA (+); CHB: 14.7%, CHC: 54.6%	178	15.7	NA	HEV higher in CHC patients (p < 0.05), speculated that HCV and HEV may share the same way of transmission
Cengiz et al. [29]	1996	NA	Samsun	C-S	Adult HD patients	72	13.9%	55	5.5	NA	p < 0.05
Cevrioglu et al. [30]	2004	2000–2002	Afyon	C-S	Pregnant women	245	12.6%	76	11.8	NA	p > 0.05
Ceylan et al. [31]	2003	NA	Diyarbakir	C-S	Agricultural workers	46	34.8%	45	4.4	NA	p < 0.05
Coursaget et al. [32]	1993	NA	Istanbul	C-S	Acute non-A non-B non-C hepatitis	18	11%	NA		NA	Probable prevalence 1–2%. Letter to a study
Koksal et al. [33]	1994	1991–1992	Diyarbakir	C-S	Acute non-A non-B hepatitis	53	73.3%	100	0	NA	
Oncu et al. [34]	2006	NA	Aydin	C-S	Pregnant women	386	7%	NA		NA	Low prevalence in high-educated
Sencan et al. [35]	2004	1999	Duzce	C-S	Children post-earthquake camps	476	4.7–17.2%	NA		NA	Duzce and Golyaka camps have significantly different rates attributed to being the first camp just after the earthquake with lower sanitation status
Uçar et al. [36]	2009	NA	Hatay	C-S	Adults HD patients	92	20.6%	NA		NA	
Yayli et al. [37]	2002	NA	Isparta	C-S	Children	340	9%	NA		NA	5–16 age range. After a hepatitis outbreak in the village, some children had symptoms and higher ALT

*CG* Control group, *C-S* cross-sectional, *NA* not available, *CHB* chronic hepatitis B, *CHC* chronic hepatitis C, *HD* hemodialysis

**Fig. 2:**
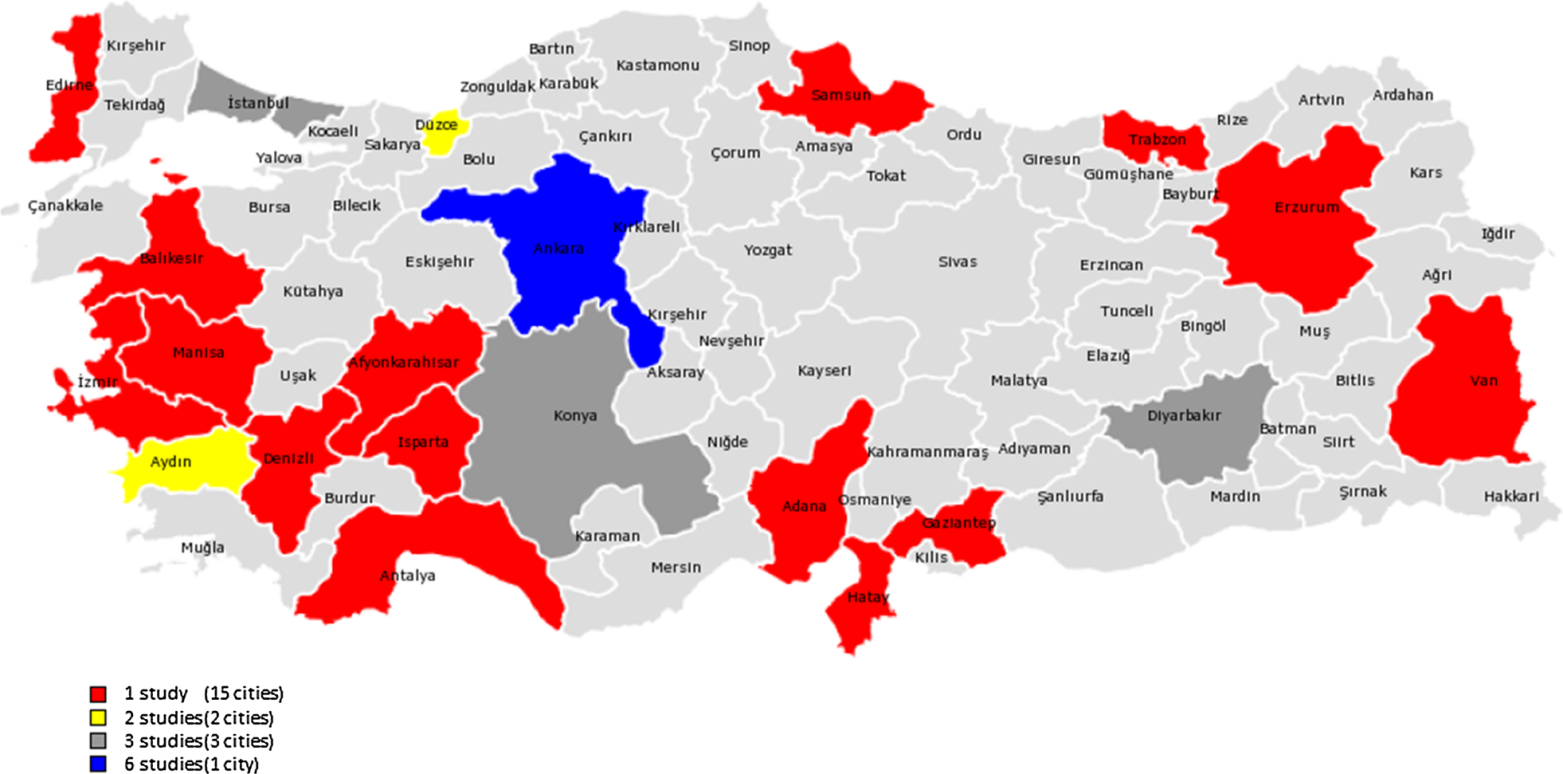
Distribution of the studies. Colors represent number of studies (total number of sites are more than actual study numbers because some studies were done in more than one city)

For the remaining six studies, two were seroprevalence studies including Turkish immigrants in Italy and the Netherlands (Table 3), and four were acute HEV infection case reports developing after travel to Turkey. The cities in which the studies were performed are given in Fig. 2.

**Table 3:** HEV infection prevalence in migrants

Authors, reference	Year	Country	Study type	Target population	Sample size	Prevalence (IgG) (%)	Power	Remarks
Chironna et al. [38]	2000	Italy	Cross sectional	Adults	368	10.3	NA	Immigrants from Turkey. No seropositives in 0–10 year-old group
Sadik et al. [39]	2004	Netherlands	Cross sectional	Adults	296	33.4	NA	Seroprevalence is similar to that in Dutch population

*NA* not available

Hepatitis E virus seroprevalence ranges from 0 to 12.4% among healthy individuals (Table 1). The prevalence was determined as 7–8% in pregnant women, 13% in chronic HBV patients, 54% in chronic HCV patients, 13.9–20.6% in patients with chronic renal failure, and ≈ 35% in agriculture workers (Table 2).

Among individuals immigrating form Turkey to Europe, HEV seroprevalence was found 10.3% in Italy [38] and 33.4% in the Netherlands (Table 3) [39]. Four patients were reported with a travel history to Turkey [from Germany (n = 1), Sweden (n = 1), and UK (n = 2)] and one died of HEV fulminant hepatitis [40–43].

## Discussion

No any outbreaks of HEV have been reported from Turkey so far. The seroprevalence of HEV depends on the region, age group, and study population. Using different ELISA kits in the diagnosis may have a role since the sensitivities of the ELISA kits are different [44, 45].

The studies were performed mainly in the big cities of Ankara and Istanbul and the study populations included blood donors and patient admitting to the hospitals with a reason other than hepatitis. For that reason, the studies give a general idea about the seroprevalence and may not provide realistic information. HEV seroprevalence is lower in children than in adults and the children lack antibodies. HEV seroprevalence is low, even zero in some pediatric series although HAV seroprevalence, another fecal–oral transmitted virus is high [10, 14, 18, 20, 22].

Similarly, a systematic review of HEV infection in children reported the seroprevalence as < 10% in children younger than 10-year old [46]. No change was detected in the seroprevalence in these children by time [13]. No any difference was detected in HEV seroprevalence in children living rural or urban areas [13, 17]. These results suggest that fecal route is not a main way of transmission or HEV transmission is low due to low fecal secretion and its low infectivity rate.

Hepatitis E virus seroprevalence increases by age in Turkey. It is higher in 3rd–4th decades and older age was determined as an independent risk factor for HEV seropositivity in a meta-analysis [10]. HEV seroprevalence differ according to the regions; being highest in the Southeastern Anatolia region and lowest in the western parts of the country [22].

Low socio-economical status may be associated with the higher seroprevalence. Seroprevalence is higher than the general population in those staying camps [35], working in agriculture and animal husbandry [31] those with chronic blood-borne infections of HBV and HCV [28], and patients with chronic renal failure given transfusions [29, 36] suggesting that more than one way of transmission may be effective.

Any study about HEV in water sources was not found in the databases. A doctoral thesis reported HEV-RNA positivity by RT-PCR in 3 out of 150 samples (drinking water, well water, swimming pool, sea water, river water, and sewage) from differing parts of the country [47]. This finding suggests a lower rate of transmission through water sources. There is a need for multi-center, well-planned epidemiologic studies searching HEV seroprevalence, ways of transmission, and risk factors in Turkey.

Turkey has been included in the endemic countries for HEV depending on two studies conducted in eastern and western parts of the country 24 years ago and far from reflecting the real situation. The seroprevalence of HEV is not exactly determined although acute hepatitis E is a reportable disease. This may be due to not using the HEV diagnostic tests commonly.

Hepatitis E virus infection may cause fulminant hepatitis and death. Turkey is among the first 10 countries of highest organ transplantation incidence in Europe (39.3 and 16.7/1 million population for kidney and liver respectively) [48]. However HEV prevalence is not known in transplanted patients or in immunosuppressed. Among individuals immigrate from Turkey to Europe; in the Netherlands, HEV seroprevalence was similar to that of the autochthonous Dutch population and another study found higher prevalence in immigrants coming from Turkey. HEV infection may challenge the immunosuppressed and those with underlying disorders especially when they travel to endemic regions. Four patients travelled to Turkey have been reported in the medical literature. Genotype 3 was detected in one case suggesting a food-borne transmission. Current data show that HEV infection related to travel to Turkey is low.

In conclusion; current review gives detailed information about HEV infection in Turkey. Previous studies suggest that Turkey is among the endemic countries of HEV. However, there are some pitfalls for the analysis of data: the studies are not powered enough to represent the whole population; they did not include immunosuppressed patients and solid organ recipients; and the prevalence of non-A non-B hepatitis was not determined. There is a need for well-designed epidemiological studies to determine HEV seroprevalence, ways of transmission, and risk factors.

## Abbreviations

HEV: hepatitis E virus
PRISMA: Preferred Reporting Items for Systematic Reviews and Meta-Analyses
SCI: Science Citation Index
WHO: World Health Organization
